# Integration of Artificial Intelligence in Dentistry: A Systematic Review of Educational and Clinical Implications

**DOI:** 10.7759/cureus.79350

**Published:** 2025-02-20

**Authors:** Bhavna Jha Kukreja, Pankaj Kukreja

**Affiliations:** 1 Periodontology, Department of Preventive Dental Sciences, College of Dentistry, Gulf Medical University, Ajman, ARE; 2 Oral and Maxillofacial Surgery, Department of Biomedical and Dental Sciences, Faculty of Dentistry, Al-Baha University, Al Aqiq Campus, Al Baha, SAU

**Keywords:** artificial intelligence, convolutional neural networks, dentistry, gamification, virtual reality

## Abstract

In dentistry, artificial intelligence (AI) revolutionized education and healthcare by providing customized, scalable, and innovative learning. Most of the review evidence focusing on the application of AI in dentistry has been published; however, this systematic review focuses on determining the knowledge, attitude, preparedness of institutions, and potential benefits of AI tools in comparison to human critical thinking by targeting studies evaluating AI’s potential in the assessment of disorders, decision-making, and simulation-oriented training. A search for relevant studies was conducted across Google Scholar, ScienceDirect, and PubMed databases from 2019 to 2024 which led to the recruitment of seven pieces of evidence demonstrating applications of AI involving, diagnostic systems, gamification tools, and virtual reality modules in dentistry, which increased engagement, and enhanced learning as well as diagnostic accuracy. However, challenges including organizational barriers, small sample sizes, and methodological differences were observed. In conclusion, for advancing the field of dentistry, AI applications demonstrate a significant contribution; however, to inculcate reliable frameworks, further investigations should focus on the conduction of longitudinal studies, restrictions to implementation, and collaboration of organizations with dental institutions.

## Introduction and background

Evidence demonstrates the influence of artificial intelligence (AI) on education and healthcare [1] as it simulates human behavior and cognition by effectively balancing the complexity of tasks and providing advanced support for executing decisions. Artificial intelligence is developed to mimic human intelligence through artificial hardware and software systems [2,3]. Hence, by utilizing the ability of AI, medical education may now offer a more flexible and effective environment for learning to evaluate and handle vast datasets [4,5].

In dentistry, AI applications enhance clinical practices and academic knowledge [4, 6, 7]. Artificial intelligence involving diagnostics, virtual simulations, and machine learning algorithms assists clinicians and academicians in training students to develop procedural skills, diagnose disorders, and strategically plan specific interventions [8,9]. For example, AI models can precisely detect abnormalities by analyzing radiographic images, allowing students to experience valuable real-world scenarios along with hands-on experience [10,11]. Moreover, it assists in monitoring the progression of patients and enables students to concentrate on their limitations by providing customized knowledge and information via tailored practicing modules. In dentistry, the utilization of AI bridges the gap between academic knowledge and practical applications along with the enhancement of the educational experience [12,13].

In dentistry, AI applications demonstrate a positive impact in terms of image diagnosis, pathology, radiography, detection of caries, electronic recordkeeping, and robotic assistance. Moreover, most of the review evidence focused on the various applications of AI in dentistry [14-16]. However, these applications are not being used routinely. Hence, this systematic review focuses on literature determining knowledge, attitude, preparedness of institutions, and potential benefits of AI tools in comparison to human critical thinking by targeting studies evaluating the capability of AI in the assessment of disorders, simulation-oriented training (technology that uses real-life scenarios allowing students to learn and practice dentistry-related skills in a safe and controlled environment), and decision-making.

## Review

Methods

Data Sources and Search Strategy

The Preferred Reporting Items for Systematic Reviews and Meta-Analyses (PRISMA) criteria were implemented for this systematic review [17]. ScienceDirect, Google Scholar, and PubMed databases were considered for literature searches covering the period from 2019 to 2024. The keywords used consisted of "Artificial Intelligence" AND "Dentistry" OR "Dental Education" to identify relevant articles.

Study Screening and Selection

The inclusion criteria consisted of evidence concentrating on the applications of AI for training and education in the field of dentistry, including knowledge, attitude, and preparedness of institutions and professionals, simulation, assessment, and virtual reality tools. Eligible studies should be observational, retrospective, cross-sectional, and randomized controlled trials (RCTs) published between 2019 and 2024. Additionally, evidence should be in English with full-text availability. However, evidence was not considered if presented protocols, editorials, or other study designs except the ones mentioned above, as well as those lacking full-text availability in English or describing inadequate knowledge regarding the preferred topic, were not considered.

The evidence was separately examined by two reviewers for their inclusion in the review. Initially, titles and abstracts were evaluated to eliminate duplicates. Next, the selected articles were re-examined to remove those not fulfilling the criteria. Lastly, to confirm eligibility, the remaining studies were assessed for full-text availability. Any discrepancies and disagreements were addressed among reviewers via mutual discussion.

Data Extraction

The authors extracted the information from the articles that comprised the study design, sample type and size, quality assessment, study objectives, methodology, outcomes, and conclusions. All the information was then reviewed and merged.

Quality Assessment

The methodological quality of the evidence was evaluated by utilizing the Mixed Methods Appraisal Tool (MMAT), as it assesses cross-sectional, mixed-method, qualitative, RCTs, and non-RCTs [18,19]. Based on the tool, the studies were rated as high, moderate, or low quality.

Data Synthesis

The critical narrative technique was used which involves synthesis using text, tables, and figures, summarizing and validating the outcomes of the evidence [18,19]. To provide a comprehensive perspective, high-quality studies, along with their biases, influencing factors, and limitations were analyzed critically. Due to the restricted quantity of appropriate evidence, a statistical or meta-analysis was not applicable. The evidence incorporated highlighted various factors and outcome measures, leading to an eminent range of heterogeneity.

Results

**Figure 1:**
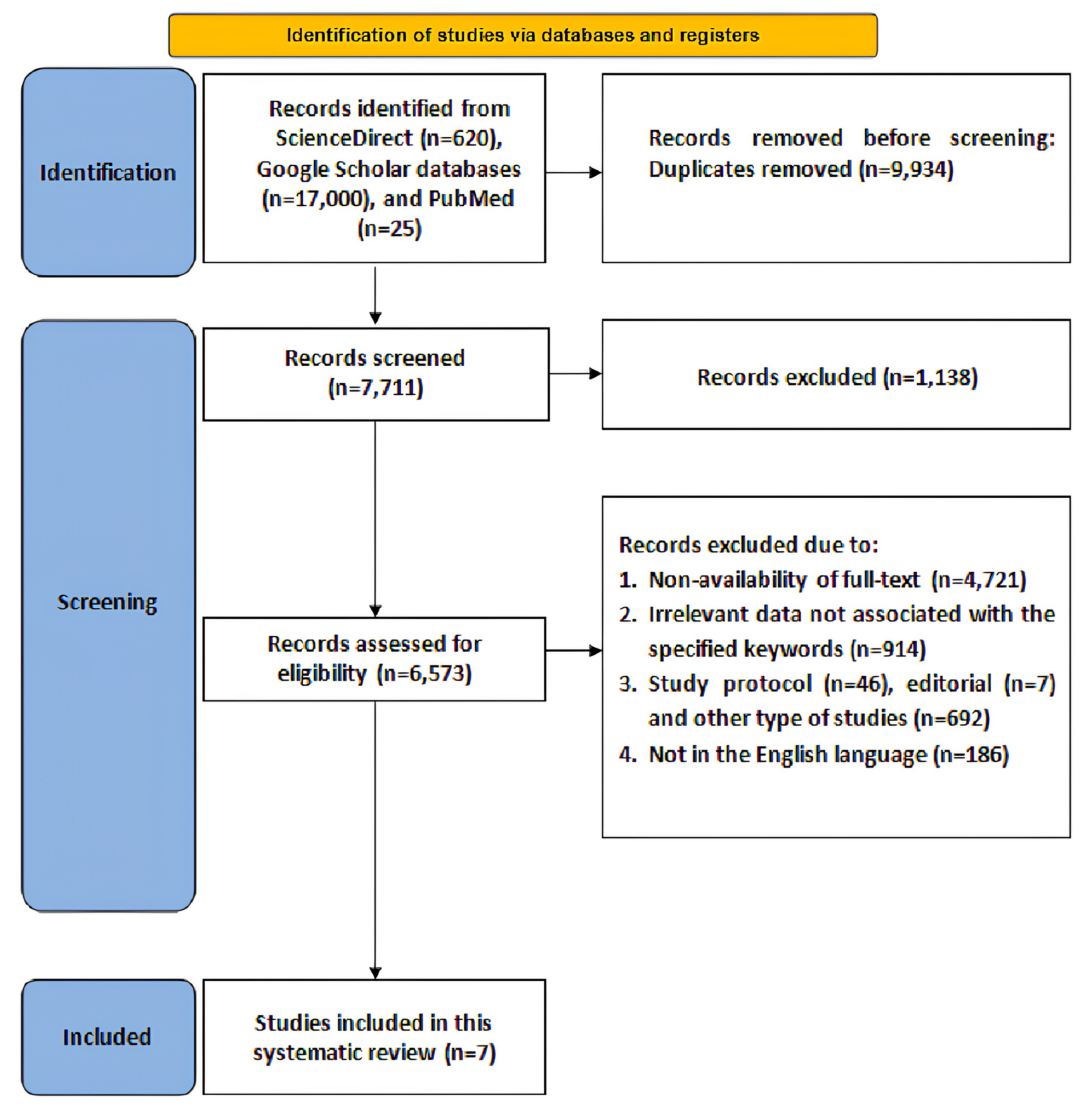
A PRISMA diagram outlining the search strategy PRISMA: Preferred Reporting Items for Systematic Reviews and Meta-Analyses

The PRISMA flow diagram is illustrated in Figure 1. Overall, 23,032 articles, including 2,303 studies from the ScienceDirect database, 3,029 from PubMed, and 17,700 from Google Scholar, were screened. After removing 14,593 duplicate articles, 8,439 articles remained for evaluation, of which 2,397 were not retrieved. Subsequently, 6,042 articles were screened for eligibility. Of these, 2,346 were irrelevant to the specified keywords, 2,769 lacked full-text availability, 652 were articles other than those mentioned in the inclusion criteria, and 268 did not have English translation available, leading to their exclusion. In total, seven studies, including RCTs, observational, and comparative studies, were included.

The details of the included studies along with the quality of the evidence are highlighted in Table 1.

**Table 1:** Demographic parameters of the included studies

Sr. No.	Study	Design	Sample size	Sample type	Quality
1.	Hamd Z et al. (2023) [4]	Cross-sectional	134	Dental professionals	High
2.	Rampf S et al. (2024) [13]	Randomized controlled trial	55	Fourth-year dental students	High
3.	Adnan K et al. (2023) [20]	Observational	200	Dental professionals	High
4.	Mahrous A et al. (2023) [21]	Randomized controlled trial	73	Dental students	High
5.	Pauwels R et al. (2021) [22]	Comparative	280	Radiograph images of 10 sockets prepared in bovine ribs	High
6.	Javed S et al. (2020) [23]	Observational	45	Primary molar teeth cases	High
7.	Patcas R et al. (2019) [24]	Observational	146	Consecutive orthognathic patients	High

The objectives, methodology, results, and conclusion of the studies are demonstrated in Table 2.

**Table 2:** A summary of the included studies

Sr. No.	Author and year	Objective	Methodology	Results	Conclusion
1.	Hamd Z et al. (2023) [4]	To assess the organizational attitude, willingness, readiness, and knowledge to incorporate artificial intelligence (AI) into dental practice.	Participants completed a validated survey to gather demographic information, perceptions, knowledge, and organizational preparation for inculcating applications of AI in dentistry.	Of the 134 respondents (78% response rate), results showed moderate to high knowledge, and enthusiasm for the integration of AI, along with a lack of training programs, highlighting the unpreparedness of organizations for the implementation of AI.	Ensuring readiness among professionals and students will enhance AI integration. Additionally, collaboration between dental institutions and societies is essential to module programs for training that bridge the knowledge gap.
2.	Rampf S et al. (2024) [13]	To assess the feasibility and potential advantages of AI integration into the educational context, including the possibility of replacing expert assessment as the gold standard.	Students were randomly categorized into either knowledge of results (KoR) feedback or elaborated feedback (eF). They had access to 16 virtual radiological example cases for eight weeks. Students were assessed on periapical radiographs for accuracy in all radiological findings detection of caries, apical periodontitis, and image quality. Moreover, the accuracy of an AI system (dentalXrai Pro 3.0) was assessed.	Among 55 students, the eF group performed significantly better in comparison to the KOR group in examining the image quality of periapical images (p = .031), detecting apical periodontitis (accuracy 0.813 ± 0.095, p = 0.011), and enamel caries (accuracy 0.840 ± 0.041, p = 0.196). The AI demonstrated near-perfect diagnostic performance, with accuracies of 96.4% for enamel caries and 98.8% for dentin caries.	Radiographic diagnostic competencies can be improved by ef, particularly in assessing apical periodontitis, and enamel caries. Hence, utilizing AI could serve as an alternative to expert labeling of radiographs.
3.	Adnan K et al. (2023) [20]	To examine the benefits and effectiveness of implementing AI and virtual reality (VR) into dental education.	The research explores AI algorithms for dental students to simulate dental procedures, enhance learning, and provide interactive training. By utilizing a quantitative method, the surveys assess participants' perceptions regarding VR systems and their impact on enhancing learning.	Virtual reality can revolutionize dental education by offering interactive and realistic simulations that provide immediate feedback, safe practice, diverse virtual patient scenarios, and personalized learning, addressing the limitations of traditional methods.	Virtual reality provides a standardized, scalable, opportunity for dentistry, elevating the proficiency of students in procedures. The study holds significant implications for professionals and institutions by providing skill acquisition, readiness for clinical practice, and learning experiences. The research encourages the development of a curriculum focusing on the implementation of AI and VR into dentistry education.
4.	Mahrous A et al. (2023) [21]	During a pre-clinical course, the study compared students' performance in removable partial denture (RPD) design, with and without using the AiDental software, while also assessing students' perceptions regarding the software.	The AiDental software provides an automatic RPD design system and a game feature comparing user designs to an ideal RPD. The study had two phases: (1) dental students (second-year) were randomly categorized into either the conventional group (n = 37) or the AiDental group (n = 36). Traditional RPD instruction was provided to both groups, with additional software access to the AiDental group (2) access to the software was provided to all, and were asked for their perceptions by offering a survey.	(1) In comparison to the conventional group, students in the AiDental group received an A or B grade. (2) Favourable perceptions were offered by the students regarding the software.	The gamification features and automated feedback of the software were well-received and positively impacted student grades describing its ability as a valuable tool to augment pre-clinical teaching.
5.	Pauwels R et al. (2021) [22]	To compare the diagnostic accuracy of human observers and convolutional neural networks (CNNs) on radiographs in diagnosing simulated periapical lesions.	Ten bovine rib sockets with three periapical defect sizes were imaged using a photostimulable storage phosphor system. A CNN model was developed with Keras-TensorFlow, and its performance was evaluated using cross-validation. The CNN's accuracy was compared to information provided by three oral radiologists.	With random validation data, the CNN achieved perfect accuracy. For radiologists, area under the receiver operating characteristic curve (AUC-ROC), specificity, and sensitivity, values were 0.75, 0.83, and 0.58, respectively whereas, when split by socket, values were 0.86, 0.88, and 0.79; and when split by filter, they were 0.93, 0.98, and 0.87 respectively.	The CNNs demonstrated the ability to diagnose periapical lesions. The pre-trained model can be elevated with clinical radiographs or larger datasets.
6.	Javed S et al. (2020) [23]	Based on pre-excavation levels of* Streptococcus mutans*, prediction of the post-excavation levels in dental caries using an iPhone operating system (iOS) application framed by using an artificial neural network (ANN) model.	Excavation of caries was conducted using a spoon excavator, polymer bur, and carbide bur. Pre- and post-excavation colony-forming units were recorded. The data were used to test develop, train, and validate various ANN models.	With an efficiency of 0.99033, the feedforward backpropagation ANN model predicts post-Streptococcus mutans. The mean absolute percentage error and the mean squared error were 4.967, and 0.2341 respectively for testing cases.	Based on pre-*Streptococcus mutans* levels and caries excavation methods, the ANN model predicts post-*Streptococcus mutans *​​​​​ levels. An iOS app was developed to assist clinicians in predicting post *Streptococcus mutans *levels, aiding caries excavation decisions, and expanding its clinical use.
7.	Patcas R et al. (2019) [24]	To use AI to evaluate the effect of orthognathic treatment on perceived age and facial attractiveness.	The pre-and post-treatment photographs of orthognathic patients were examined. Facial attractiveness along with apparent age was assessed using CNNs trained on over images for age (0.5 million) and for attractiveness (17 million ratings).	Orthognathic treatment also improved attractiveness in 74.7% of patients (p<0.001), with greater effects following lower jaw surgery. Additionally, the algorithms showed that 66.4% of patients experienced improved appearance, with an average age reduction of nearly one year (p=0.002), especially after profile-altering surgery.	This study indicates that AI can effectively assess perceived age and facial attractiveness in orthognathic patients.

Educational Perspective

Hamd Z et al. (2023) assessed the organizational attitude, willingness, readiness, and knowledge to incorporate AI into dental practice and found that 78% of results showed moderate to high knowledge and enthusiasm for the integration of AI, but a lack of training programs highlighted the unpreparedness of organizations for the implementation of AI. Hence, the study concluded that ensuring readiness among professionals and students will enhance AI integration. Additionally, collaboration between dental institutions and societies is essential to module programs for training that bridge the knowledge gap [4]. Moreover, Rampf S et al. (2024) assessed the feasibility and potential advantages of AI integration into the educational context, including the possibility of replacing expert assessment as the gold standard. The results showed that AI demonstrated near-perfect diagnostic performance, with accuracies of 96.4% for enamel caries and 98.8% for dentin caries. Hence, the study concluded that radiographic diagnostic competencies can be improved by elaborated feedback, particularly in assessing apical periodontitis and enamel caries. Hence, utilizing AI could serve as an alternative to expert labeling of radiographs [13]. Additionally, Adnan K. et al. (2023) examined the benefits and effectiveness of implementing AI and virtual reality (VR) into dental education. The outcomes demonstrated that VR can revolutionize dental education by offering interactive and realistic simulations that provide immediate feedback, safe practice, diverse virtual patient scenarios, and personalized learning, addressing the limitations of traditional methods. Hence, the study concluded that VR provides a standardized, scalable, opportunity for dentistry, elevating the proficiency of students in procedures. The study holds significant implications for professionals and institutions by providing skill acquisition, readiness for clinical practice, and learning experiences. The research encourages the development of a curriculum focusing on the implementation of AI and VR into dentistry education [20]. Furthermore, during a pre-clinical course, Mahrous A et al. (2023) compared students' performance in removable partial denture (RPD) design, with and without using the AiDental software, while also assessing students' perceptions regarding the software [21]. The results showed that in comparison to the conventional group, students in the AiDental group received an A or B grade, and favorable perceptions were offered by the students regarding the software. Hence, the study concluded that the gamification features and automated feedback of the software were well-received and positively impacted student grades, describing its ability as a valuable tool to augment pre-clinical teaching.

Clinical Perspective

Pauwels R et al. (2021) compared the diagnostic accuracy of human observers and convolutional neural networks (CNNs) on radiographs in diagnosing simulated periapical lesions. With random validation data, the CNN achieved perfect accuracy. For radiologists, area under the receiver operating characteristic curve (AUC-ROC), specificity, and sensitivity values were 0.75, 0.83, and 0.58, respectively, whereas, when split by socket, values were 0.86, 0.88, and 0.79; and when split by filter, they were 0.93, 0.98, and 0.87, respectively. Hence, the study concluded that CNNs demonstrated the ability to diagnose periapical lesions. The pre-trained model can be elevated with clinical radiographs or larger datasets [22]. Additionally, based on pre-excavation levels of *Streptococcus mutans*, Javed S et al. (2020) predicted the post-excavation levels in dental caries using an iPhone operating software (iOS) application framed by using an artificial neural network (ANN) model. With an efficiency of 0.99033, the feedforward backpropagation ANN model predicts post-*Streptococcus mutans* levels. The mean absolute percentage error and the mean squared error were 4.967 and 0.2341, respectively, for testing cases. Hence, the study concluded that based on pre-*Streptococcus mutans* levels and caries excavation methods, the ANN model predicts post-*Streptococcus mutans* levels. An iOS app was developed to assist clinicians in predicting post-*Streptococcus mutans* levels, aiding caries excavation decisions, and expanding its clinical use [23]. Furthermore, Patcas R et al. (2019) used AI to evaluate the effect of orthognathic treatment on perceived age and facial attractiveness and concluded that AI can effectively assess perceived age and facial attractiveness in orthognathic patients [24]. 

Discussion

This systematic review highlights the significant potential transformation of the field of dentistry through the application of AI. Across the included studies, AI applications demonstrated their ability to improve learning outcomes, enhance diagnostic accuracy, and provide personalized education experiences. For instance, Rampf et al. (2024) highlighted how AI feedback mechanisms outperformed traditional methods in enhancing students' radiographic diagnostic competencies. This implies that AI can be a practical alternative to expert-led assessments in specific educational settings [13]. eF, widely studied in computer-based assessments, has shown effectiveness in enhancing diagnostic skills through a case-based worked example approach in medical education [25]. This study is the first to compare elaborated feedback (eF) and knowledge of results (KoR) feedback in dental education, focusing on radiographical diagnostic competencies. Additionally, it explores the potential of AI applications in dental training, emphasizing the growing relevance of integrating AI into dental curricula amidst its rising adoption in healthcare [13]. Elaborated feedback significantly enhanced diagnostic accuracy, particularly in identifying enamel caries and apical periodontitis. Comparable improvements in detecting enamel caries have also been observed in previous studies where dentists utilized AI applications for support. Both teacher-provided eF and AI-based feedback seem effective in enhancing diagnostic competencies. However, future research should investigate students' treatment decisions following radiographic diagnostics, as increased sensitivity in detecting enamel caries may risk leading to overtreatment [25].

Pauwels et al. (2021) further demonstrated the impact of AI on enhancing clinical decisions and revealed that CNNs surpassed human observers in detecting periapical lesions on radiographs. Such findings highlight the potential of AI to enhance both student training and clinical practice by offering accurate diagnostic support [22]. Previous diagnostic studies have concentrated on periapical evaluation, dental caries, and periodontal assessment, consistently demonstrating high diagnostic accuracy following training [26,27]. A previous study on CNN-based periapical assessment reported an accuracy of 0.88 through transfer learning; however, it did not include a comparison with human observers [27]. Additionally, data labeling was carried out by dentists and radiologists [27]. In radiologic AI research, the prevailing trend suggests that when reliable ground truth data is available for labeling, trained CNNs can match or even exceed the diagnostic performance of experienced clinicians.

Another significant example is the study by Adnan et al. (2023), which highlighted the immersive capability of VR. The study concluded that the participants narrated positive feedback about the interactive training modules that replicated realistic dental procedures. These systems overcame the limitations of traditional dental education, such as uneven clinical exposure, by offering standardized yet adaptable learning environments [20]. Most participants concurred that these systems improve learning outcomes by offering realistic scenarios and precise guidance. The integration of AI-generated feedback within the virtual environment further enriched the learning experience. Participants highlighted the value of this feedback in pinpointing areas for improvement, enabling focused practice and skill enhancement [20]. Participants reported high levels of satisfaction with the realism of virtual scenarios, AI-generated feedback, and the user-friendly design of the systems. These findings suggest that AI-driven VR systems are well-regarded by both students and educators, highlighting their potential for broader implementation in dental education. Additionally, the study indicates that these systems are perceived as moderately to highly accessible [20]. Participants emphasized the availability of necessary hardware and software, along with the ease of use and user-friendly design of the systems. This accessibility plays a vital role in ensuring that dental students, regardless of their location or resources, can benefit from these educational tools. Additionally, participants reported enhancements in knowledge retention, practical skills, confidence in performing dental procedures, and comprehension of complex dental concepts [20].

Similarly, Mahrous et al. (2023) showed how gamified AI tools, like AiDental, notably improved student performance in pre-clinical courses. The gamification approach not only boosted grades but also fostered greater student engagement and critical thinking [21]. Borit et al. implemented a board game approach in which students engaged by formulating questions that their peers answered. The findings revealed that this game-based learning strategy enhanced students' attentiveness, motivation, and overall enjoyment of the learning process. The AiDental software holds clinical potential as a supportive tool for decision-making in practice [28]. It can also serve to standardize and calibrate designs across different philosophies while highlighting the distinctions between them. Chen et al. developed a case-based decision tree capable of generating text-based recommendations for major connector and clasp assembly selection for RPDs based on the pattern of missing teeth. However, the design did not account for abutment tooth characteristics, such as undercut depth and location, which did not influence the recommendations [29].

In addition, Javed et al. (2020) showcased the value of an AI-based iOS application in predicting the outcomes of dental caries treatment, further illustrating the potential of AI in clinical decisions [23]. Heckerling et al. explored three different architectures for predicting community-acquired pneumonia and discovered that a network with a single hidden layer performed as accurately as one with two hidden layers [30]. A neural network was created with four input nodes, a hidden layer consisting of four nodes, and a single output node to forecast the success rate of weaning patients from a mechanical ventilator [31]. Predicting *post-Streptococcus mutans* levels before caries excavation using various methods helps in choosing the most suitable excavation technique, leading to a caries-free cavity preparation for restoration. Mobile applications have become a widely used tool for learning and diagnostics in dentistry [32]. Numerous dental apps are accessible on the Google Play Store and iTunes. However, the credibility of an app is determined by the reliable research provided by its developer [33]. In the present study, data with an uncertainty of under 2.5% were obtained during the experimental trials, and a highly accurate model with a prediction accuracy of 99% was developed for forecasting post-*Streptococcus mutans* levels. Challenges related to the accessibility and implementation of the currently developed ANN model by clinicians worldwide, whether or not they have knowledge of ANN modeling, need to be addressed. Therefore, the ANN model was integrated into an app, enabling clinicians to effortlessly predict post-*Streptococcus mutans* levels. The PSm iOS app was designed with user-friendliness in mind, so when predicting post-*Streptococcus mutans*, clinicians only need to input the pre-*Streptococcus mutans* value and the caries excavation method used.

The studies included demonstrate several strengths. First, the studies utilized a range of AI technologies, such as CNN [23], gamification tools [21], and virtual reality systems [20], demonstrating applications of AI in dentistry. Secondly, many employed strong methodological designs, such as RCTs [13,21] and observational studies with clearly defined objectives [4,20]. These designs facilitated thorough analysis and provided reliable conclusions. Furthermore, the use of validated assessment tools, including AI feedback systems and ANN iOS applications, strengthened the credibility and relevance of the findings [23]. However, a key limitation in the studies was the absence of longitudinal data regarding the long-term impact of AI in dentistry. Another challenge was the limitation to the uniformity of the findings due to the small sample [13, 23]. Moreover, Hamd et al. (2023) identified deficiencies in organizational preparedness and the necessity for targeted training programs, indicating that addressing infrastructural and educational barriers is crucial for maximizing the potential of AI [4].

Additionally, a major challenge is data dependency, as AI requires large, annotated, and high-quality datasets for effective training. However, the scarcity of such datasets in dentistry hinders the implementation and development of AI systems [22]. Furthermore, regarding the protection of patient privacy and data security, legal and ethical challenges present significant concerns. Furthermore, complications related to the broader adoption should be addressed by implementing comprehensive regulatory frameworks for the integration of AI [5]. Moreover, as AI systems often require advanced computing infrastructure, technological limitations are also a key factor. The ability and progress of the AI models are completely based on the interpretability and reliability of algorithms, which can be challenging to acquire [34]. This highlights the need for human critical thinking, as more dependency on AI could lead to complications among practitioners. Hence, it is stated that AI can assist clinicians and academicians but cannot replace human critical thinking [2]. Finally, lack of awareness, mistrust, and adoption continues to be a barrier due to inadequate knowledge and training. This depicts the need for targeted educational programs to build competency and trust in adapting and utilizing AI models [4,35,36].

Strengths and Limitations

The review incorporates insights from diverse global studies. It highlights limitations by providing active and practical suggestions to overcome them, developing a progressive environment. However, the limitation observed was the consideration of investigations available in only English, leading to the elimination of relevant articles in other languages. Another limitation involved was coverage of articles published from 2019 to 2024 which restricted the inclusion of foundational articles.

Recommendations and Future Directions

Multi-centre RCTs should be performed with larger samples to ensure the generalizability of the outcomes. Additionally, the long-term impact of AI applications in dentistry should be examined to assess effectiveness and sustainability over time for which longitudinal studies should be focused. The development and sharing of high-quality datasets should be encouraged for AI model training related to dentistry. Educational programs for AI literacy among clinicians, students, and academic institutions must be targeted to enhance learning and trust in AI. Moreover, partnerships or collaborations across stakeholders should be encouraged to streamline the integration of AI in dentistry. Future directions should focus on customized learning modules based on student performance and feedback, the development of regulatory frameworks for AI applications in dentistry to ensure efficacy, conduction of studies in developing areas to address the challenges associated with AI adoption. Furthermore, frameworks for legal and ethical concerns should be focused on maintaining the privacy of the patient.

## Conclusions

This systematic review highlights the impact of applications of AI in dentistry by improving clinical decisions, diagnostic accuracy, and learning approaches. Machine learning models gamification and VR-enabled customized learning by effectively addressing conventional barriers. However, impactful integration is possible by overcoming challenges such as technological and ethical barriers, small samples, and organizational preparedness. Further investigations should target conducting long-term studies, providing specific training, and emerging vast datasets. Collaboration among professionals, technology developers, organizations, and academic institutions is crucial for integrating AI in dentistry.
